# The Narrative Coherence of Autistic Children’s Accounts of an Experienced Event in Response to Different Interviewer Prompts: A Longitudinal Study

**DOI:** 10.1007/s10803-024-06675-x

**Published:** 2024-12-28

**Authors:** Telma Sousa Almeida, Fuming Yang, Heying Zhang, Michael E. Lamb

**Affiliations:** 1 Department of Psychology, University of Cambridge, Cambridge, UK; 2 William James Center for Research, ISPA-Instituto Universitário, Rua Jardim do Tabaco, 34, Lisbon, 1149-041 Portugal; 3 Department of Molecular and Cellular Biology, Harvard University, Cambridge, USA; 4 Division of Science & Technology, BNU-HKBU United International College, Zhuhai, China

**Keywords:** Autism, Narrative coherence, Delay, NICHD and eyewitness testimony

## Abstract

**Purpose:**

This study explored the narrative coherence of the accounts of an experienced event produced by autistic and neurotypical children (ages 6–15 years) after delays of two weeks and two months.

**Methods:**

The sample comprised 27 autistic children and 32 neurotypical peers, who were interviewed about the event using the Revised National Institute of Child Health and Human Development (NICHD) Investigative Interview Protocol. The study focused on assessing the narrative coherence of children’s reports, emphasizing key story grammar elements and temporal features in their narratives.

**Results:**

Results revealed that, over time, both autistic and neurotypical children showed a decrease in narrative coherence. Autistic children, particularly those who were cognitively and verbally able, demonstrated the ability to convey their experiences coherently, with performances comparable to those of their neurotypical peers. Interviewer prompts differentially influenced the narrative coherence of autistic and non-autistic children’s accounts.

**Conclusion:**

This research showed that, when questioned appropriately, cognitively and verbally able autistic children can effectively communicate their personal experiences, even after significant delays.

In many criminal investigations, physical evidence of the abuse may be absent, and case outcomes frequently rely on children’s eyewitness testimony (Milne & Bull, 1999; Pipe et al., 2007; Wells et al., 2006). That testimony is most likely to be deemed credible when it is clear and logically organized (Brown et al., 2018a; Henry et al., 2020; Szojka et al., 2020; VanMeter et al., 2023). However, some autistic children have unique cognitive and socioemotional characteristics (e.g., Hajri et al., 2022; Karalunas et al., 2018; Löytömäki et al., 2023; Neuhaus et al., 2019) that both heighten their susceptibility to victimization (Chan & Lam, 2016; Frederick et al., 2019; Hellström, 2019; Hwang et al., 2020) and affect their ability to recall and narrate personal experiences coherently. This study sought to explore how narrative coherence—defined as the logical organization and clarity of a story—evolves in autistic children over time and how it is affected by the way they are questioned.

## Autism, Memory, and Testimony

Individuals on the autism spectrum are disproportionately likely to be victimized (Brown et al., 2017; Cazalis et al., 2022; Collins et al., 2022; Hoover & Kaufman, 2018; Weiss & Fardella, 2018) and may encounter difficulties recognizing or reporting abusive situations, in part due to cognitive and communicative differences (Harrell, 2021; Hwang et al., 2018; Prosecution Service of Georgia, 2022; Trundle et al., 2022; Turcotte et al., 2018) that can be exacerbated by the long delays commonly encountered in legal proceedings (e.g., Goodman et al., 1992; Peixoto et al., 2017) which further erode memory retrieval in children, including those on the autism spectrum (Almeida et al., 2019a; Brown et al., 2012, 2015).

The communication difficulties associated with autism can affect how children’s testimony is perceived (Lim et al., 2022; Steel, 2016). Clear narratives enable legal practitioners to better understand the sequence of events and the actions of alleged perpetrators, facilitating more accurate case assessments (VanMeter et al., 2023), including assessments of credibility (Dritschel et al., 2010; Feltis et al., 2010; Murfett et al., 2008; Szojka et al., 2017, 2020). It is not clear whether autism affects the ability to narrate events coherently although Henry et al.(2020) found no significant differences between the overall coherence of narratives provided by autistic and non-autistic children.

However, even when narratives appear similarly coherent, the underlying cognitive and communicative processes may differ, affecting how these narratives are perceived in forensic settings. Both the Central Coherence Theory and Theory of Mind (ToM) frameworks suggest that autistic children may provide less coherent narratives than non-autistic children. Central Coherence Theory (Baron-Cohen et al., 1985) posits that autistic people often focus on details at the expense of the broader context, which may lead to narratives that are rich in specifics but lack overall coherence or flow (Szojka et al., 2017, 2020). For instance, Brown et al. (2018) observed that while autistic children could generate detail-rich narratives, these were often not coherently organized, raising questions about how their memory and narrative skills interact. Some autistic children also struggle with ToM tasks (Abell et al., 2000; Baron-Cohen, 1995; Baron-Cohen et al., 1985; Happé, 1994; Kaland et al., 2005) and these deficits further complicate narrative construction by affecting the ability to understand, infer, and integrate into narratives the thoughts, feelings, and motivations of others.

Linguistic abilities also play a crucial role in narrative coherence. Autistic children’s narratives tend to be semantically poorer and more reliant on experimenter guidance (King et al., 2013; Losh & Capps, 2003; Losh & Gordon, 2014); even autistic adults have difficulties integrating complex syntax and explaining characters’ intentions in their narratives (Lee et al., 2018). These linguistic challenges contribute to struggles with key elements of narrative coherence, such as logical flow, chronological order, and causal explanation—especially when external structural support is not provided (Losh & Capps, 2003). Further, even preschool-aged autistic children with advanced language skills can have difficulty grasping and processing complex language (Cleland et al., 2010; Koning & Magill-Evans, 2001; Rapin et al., 2009; Rapin & Dunn, 2003; Saalasti et al., 2008; Shriberg et al., 2001; Williams et al., 2006). These challenges persist as children grow older, even as articulation and syntax improve, with comprehension and semantic difficulties continuing to make it harder for some autistic children to provide coherent narratives without structured support (Condouris & Tager-Flusberg, 2003; Kjelgaard & Tager-Flusberg, 2001).

Indeed, without tailored support, some autistic children tend to recall less information about events than do their non-autistic peers, especially when asked to provide spontaneous, detailed accounts (Almeida et al., 2019a; Bruck et al., 2007; Henry et al., 2017a; Mattison et al., 2015, 2016; McCrory et al., 2007a). However, when provided with appropriate support in the form of questioning techniques that accommodate their cognitive styles, autistic children can recall information accurately and reliably (Bruck et al., 2007; Henry et al., 2017a; Henry, Messer, Henry et al., 2017a, b). However, what about the coherence of the narratives in which that information is embedded?

## Narrative Coherence and the Influence of Delay and Interview Prompts

Narrative macrostructure refers to key story grammar elements, such as setting, initiating events, and character responses (Stein & Glenn, 1979) whereas coherence refers to how logically and chronologically these elements are organized to effectively communicate experiences (Diehl et al., 2006; Reese et al., 2011; Stein & Nezworski, 1978). Some autistic children produce narratives that include fewer and less complex story grammar elements (Peristeri et al., 2017; Sangani et al., 2023) and this study focused on how these elements are integrated into coherent and comprehensible accounts and how that varied depending on the length of delays since the events occurred.

Early studies (Stein & Glenn, 1979; Stein & Nezworski, 1978) suggested that higher-order narrative structuring, such as coherence, played a critical role in the accuracy of story recall but later research has yielded mixed results. Bruck et al. (2002) and Kulkofsky et al. (2008) found no significant correlation between narrative quality and the amount or accuracy of information recalled whereas other research suggests a more nuanced relationship that is particularly relevant in forensic testimony settings. For example, Klemfuss and Wang (2017) reported that children with stronger narrative skills often produced more elaborate and engaging accounts, albeit characterized by more inaccuracies. Kulkofsky and Klemfuss (2008a) found that, regardless of age, children with high-quality narrative skills specific to an event were better able to resist suggestive questioning, reinforcing the notion that strong event-specific narrative skills support memory accuracy. However, children with high general narrative abilities (not specific to the target event) showed increased susceptibility to suggestive pressure, highlighting the complex and context-dependent relationship between narrative skills and memory accuracy.

A meta-analysis by Baixauli et al. (2016) highlighted significant differences between the narratives created by autistic and non-autistic children with respect to story length, syntactic complexity, causal explanations, and the use of ambiguous pronouns. Autistic children’s narratives tended to be highly detailed but lacked a coherent overall structure, which is critical in forensic contexts (Banney et al., 2015; Barnes & Jacobs, 2013; Capps et al., 2000; Goldman, 2008; King et al., 2013; Lee et al., 2018; Losh & Capps, 2003; McCabe et al., 2013; Norbury & Bishop, 2003; Pearlman-Avnion & Eviatar, 2002). However, some studies have found no significant differences in narrative coherence between autistic and non-autistic children (e.g., Henry et al., 2020; Norbury & Bishop, 2003; Young et al., 2005), suggesting that methodological differences across studies may contribute to the variability in findings.

Researchers have indeed employed various methods, ranging from construction and demonstration tasks to storytelling using ambiguous pictures (King et al., 2013; Lee et al., 2018; Losh & Capps, 2003; Norbury & Bishop, 2003). These diverse approaches have led to variability in observed narrative performance. For instance, structured tasks such as picture-based storytelling tend to yield more similar levels of performances by autistic and non-autistic children (Goldman, 2008; Losh & Gordon, 2014), whereas open-ended tasks often reveal more pronounced differences in narrative coherence (King et al., 2013; Losh & Capps, 2003). Such differences must obviously be taken into account when generalizing from analogue studies to forensic contexts in which the reliability of testimony is of critical importance.

The present study was designed to assess and compare the coherence of narratives produced by autistic children and their non-autistic peers about a complex experienced event under different recall delays and questioning styles. By applying story grammar frameworks, we aimed to provide a more comprehensive understanding of how autistic children and their non-autistic peers organize and present coherent narratives. This methodological approach was designed to highlight both the strengths and challenges autistic children face, offering valuable insights into how different recall conditions and interviewer prompts influence their ability to produce coherent, structured, and reliable accounts.

Research consistently shows that the quality of information provided by children in investigative interviews is closely tied to the style of questioning, with the formulation of questions and the level of interviewer input significantly affecting both the accuracy and richness of children’s reports (Lamb et al., 2011, 2015, 2018). This is especially relevant in forensic contexts, where narrative coherence, defined by logical organization and clarity, plays a key role in assessing the credibility of testimonies. While prior research has explored how autistic children recall events shortly after they occur (Henry et al., 2017a, b; Mattison et al., 2015, 2016) or after prolonged delays (Almeida et al., 2019b), fewer studies have examined the long-term coherence of their narratives in response to different interview prompts. Therefore, understanding how question format and time delays affect the coherence of autistic children’s narratives should inform forensic interviewing practices.

Parte superior do formulário.

Parte inferior do formulário.

*The Current Study*.

This study was designed to enhance our understanding of how autistic children articulate their experiences, particularly in the context of investigative interviews. We aimed to investigate the effects of time delays and interviewer prompts on the narrative coherence of autistic children’s accounts compared to those provided by non-autistic peers. Specifically, we assessed how these children recall events after two delays—two weeks and two months—and examined the influence of different questioning techniques on the coherence of their narratives. The two-week delay captured how memory retention operates over shorter periods, relevant to the early stages of forensic investigations when children are typically interviewed shortly after an incident. In contrast, the two-month delay assessed how memory retrieval and narrative coherence held up over longer periods, a crucial factor when significant time has passed, as often happens before legal proceedings. By comparing these time intervals, our study was designed to explore differences or similarities in how autistic and non-autistic children encode, retain, and retrieve memories, offering practical insights for refining investigative interview techniques to accommodate different recall scenarios.

In addition to examining time delays, it is crucial to consider the role of different questioning techniques in shaping children’s narrative responses. The type of question asked—whether open-ended, specific cued recall, or close-ended recognition prompts—significantly affects the coherence and content of children’s narratives (Almeida et al., 2019b; Brown & Lamb, 2015; Feltis et al., 2010). Moreover, in assessing narrative abilities, potential confounds between the scope of the questions (open versus focused) and the nature of the task (recall versus recognition) must be considered, as autistic children’s responses might vary depending on these variables. This variability may affect both the coherence and richness of their narratives. Some autistic children, due to varied communication preferences and cognitive processing, may respond differently to the types of questions they are asked. Therefore, we examined how these different types of interviewer prompts affected narrative coherence in both autistic and non-autistic children, addressing a critical gap in the literature regarding the impact of questioning techniques on the quality of children’s testimonies.

In our study, we used the Revised National Institute of Child Health and Human Development (NICHD) Investigative Interview Protocol (Lamb et al., 2018), a widely-used framework for obtaining reliable testimony from children. Originally developed to enhance the quality of information gathered during forensic interviews with children, the Revised NICHD Protocol emphasizes the use of open-ended prompts and a narrative-based approach to minimize interviewer bias and maximize the accuracy of children’s reports. Given the unique communication styles and cognitive challenges faced by autistic children, it is crucial to assess whether this Protocol, or adaptations of it, can be used effectively with these children. In this study, we aimed to explore how different questioning techniques, including the use of open-ended versus specific and recognition-based prompts, influence the coherence of autistic children’s narratives. The findings should help identify the best practices for interviewing autistic children in forensic contexts, providing insight into the optimal conditions for eliciting coherent and reliable accounts.

Our hypotheses were informed by existing literature on narrative coherence and memory retention in children. First, we hypothesized that autistic children would produce narratives as coherent as their non-autistic peers when provided with appropriate questioning support, reflecting the potential for both groups to offer reliable and detailed information under the right conditions (Bruck et al., 2007; Henry et al., 2017a). Second, we anticipated that narrative coherence would decline over time for both autistic and non-autistic children, in line with previous research suggesting that memory detail and accuracy tend to diminish as delays increase (Almeida et al., 2019a; Klemfuss & Wang, 2017). Additionally, we hypothesized that open-ended recall prompts would elicit more coherent narratives than directive or option-posing questions for both groups, based on the established effectiveness of such prompts in previous studies (Almeida et al., 2019b; Lamb et al., 2018).

## Method

### Sample

The data for this study were derived from interviews conducted during a prior research project (author, year; blinded). Our sample comprised fifty-nine children aged between 6 and 15 years (*M* = 9 years, 9 months), including 27 autistic children (23 males, 4 females) and 32 non-autistic children (18 males, 14 females). All participants, or their legal representatives, provided informed consent, adhering to the National Health Service Research Ethics Committee’s protocol.

Autistic children were recruited from the health service. Each autistic participant had a formal diagnosis from a qualified clinical expert, meeting the DSM-V criteria for autism spectrum disorder (American Psychiatric Association, 2013), as confirmed by the Autism Diagnostic Observation Schedule, Second Edition (ADOS-2; cut-off point of seven or eight) and the Autism Diagnostic Interview, Revised. These autistic children, who had verbal quotients of 85 or above and full-scale IQs of 90 or above as measured using the Wechsler Intelligence Scale for Children-Third Edition, were referred for participation post-diagnosis. This assessment approach was chosen due to the significant variability in cognitive functioning within the autistic population and was crucial for ensuring a reliable evaluation of intellectual and linguistic abilities.

The non-autistic group, recruited through local mainstream schools, comprised children with no known psychiatric, developmental, or neurological disorders as reported by their parents/caregivers. They were included in the study if they scored below the autism spectrum cut-off on the ADOS-2 (i.e., six or less), serving as an initial indicator of typical development. Although we were unable to conduct IQ testing for the non-autistic group due to practical constraints, we ensured a baseline cognitive functioning level comparable to the autistic group though parental reports regarding their children’s academic performance, cognitive, and social functioning. To further support the comparability of the groups, we relied on indirect indicators such as the type of school attended and the absence of reported learning difficulties, based on parental feedback, to infer a baseline level of cognitive functioning. This approach, while not as direct as standardized testing, aimed to create groups that were comparable based on the available information. This criterion was a necessary adaptation to balance thoroughness with feasibility in our study. Furthermore, both groups were closely matched for chronological age, a factor often correlated with cognitive development, with independent *t*-tests confirming no significant difference in age distribution between them (autistic: *M* = 10.6, *SD* = 3.0, range = 6–15; non-autistic children: *M* = 9.4, *SD* = 2.7, range = 6–15).

### Materials and Procedure

In this study, the Autism Diagnostic Observation Schedule, Second Edition (ADOS-2) activities were employed as the target event. The choice of ADOS-2, a standardized, interactive assessment tool, was deliberate. ADOS-2 activities are designed to simulate naturalistic social interactions and are thus reflective of typical children’s experiences, providing a neutral and comparable context for both autistic and non-autistic groups. This approach ensured that our findings were grounded in a universally applicable, ecologically valid framework. ADOS-2’s structure and content are tailored to elicit natural responses in varied social and communicative contexts, making it an ideal tool for observing and comparing narrative abilities in a controlled setting. We carefully observed and recorded how both groups engaged with the ADOS-2 tasks, paying close attention to their social interactions, responses to prompts, and overall engagement, to ensure that any qualitative differences in these behaviours did not confound the results. Furthermore, we recognize that the dynamics of memory retrieval could vary between autistic and non-autistic children. To account for this, our analysis methodically examined how each group processed and recalled the ADOS-2 experience, ensuring a balanced and fair comparison.

All children participated in the complete set of ADOS-2 activities, which included engaging interactive stimulus materials. Specifically, the event-to-be-remembered for the interviews encompassed: a construction task, a make-believe play, a joint interactive play, a demonstration task, the description of a picture, telling of a story from a book, telling of a story depicted in cartoons, conversations about something that happened to the child in the past, questions about a variety of topics, a break, and the creation of a story using objects provided. Following their participation, each child was systematically interviewed about their experience with the entire range of activities. For autistic children, a qualified psychiatrist conducted the ADOS-2 as part of the child’s diagnostic process, independently from the research study. This session occurred two weeks before children took part in the study. Non-autistic children experienced the activities included in the ADOS-2 as part of the research study. These sessions were conducted by a member of the research team, with prior ADOS-2 training. For a more detailed description of the activities, please see (author, year; blinded; Lord et al., 2012).

After participating in the to-be-remembered event, which encompassed multiple tasks and activities as part of the ADOS-2, children were interviewed after two weeks and again after two months. During these interviews, they were explicitly asked to recount and elaborate on each activity they had engaged in, aiming to capture detailed narrative accounts of their experiences across the entirety of the session, treating the full ADOS-2 administration as a single, coherent event with multiple components. One-way analyses of variance (ANOVAs) confirmed that the groups did not differ significantly with respect to the delay (in days) between the event and the first interview, *F*(1, 57) = 1.19, *p* = .281 (autistic: *M* = 12.2, *SD* = 3.4, 95% CI [*10.84*,* 13.53*]; non-autistic: *M* = 11.1, *SD* = 4.4, 95% CI [*9.50*,* 12.63*]); and between the event and the second interview, *F*(1, 57) = 3.95, *p* = .052 (autistic: *M* = 65.0, *SD* = 13.6, 95% CI [*59.62*,* 70.38*]; non-autistic: *M* = 73.8, *SD* = 19.5, 95% CI [*66.82*,* 80.86*]).

Both interviews were conducted using the Revised NICHD Protocol developed by Lamb and colleagues (see Lamb et al., 2018 for a full description of the Protocol), and administered by licensed forensic psychologists trained in the NICHD Protocol. All interviews, at both time points, comprised the same phases in the same order, as follows: (1) greet; (2) rapport (3) ground rules, truth-and-lie exercise; (4) substantive recall part of the interview (i.e., interviewers’ questions and children’s responses regarding the investigated event); and (5) closure. This research focused on the information elicited during the *substantive portion of the interviews*, which began with an open prompt inviting children to provide as much information as they could remember about the event. Children were then encouraged to provide further details about the information that they had just reported using a series of follow-up open-ended prompts. More focused questions, such as yes/no and forced-choice option-posing prompts, were avoided, but occasionally used if needed to clear up ambiguity and these were followed by open prompts. Once the child had finished speaking and was waiting for the next prompt, they were once again asked: “Is there anything else you remember?”. This prompt was repeatedly asked until the child could not offer further information.

### Data Coding

All interviews were video-recorded and transcribed verbatim. Coding focused on information that pertained to the target event (i.e., the substantive portion of the interview), therefore excluding any introductory exchanges at the beginning of the interview, attempts to establish rapport with the child, and attempts at the end of the interview to discuss neutral topics.

### Interviewer Utterance Types

For the purposes of the present study, interviewer utterances were coded using the NICHD Interview Coding Scheme (see Lamb et al., 2008 for a full review) as invitations, directive or option-posing prompts and the total number of each type of utterances was recorded for each child. Each question type is described below.

*Invitations* referred to open-ended utterances using questions, statements, imperatives, or contextual cues to elicit narrative free-recall responses. These did not restrict the child’s focus except in a general sense. Invitations could refocus the child’s attention on previously mentioned details, using them as contextual cues to elicit narrative free-recall responses or could follow-up on information just mentioned or request additional free-recall elaboration about details previously mentioned. Invitations included the following variations: general invitation, such as utterances asking about a whole activity or about one of multiple activities (e.g., Tell me everything that happened from the beginning to the end; Tell me everything about the first/ last/ best remembered [child’s label] activity); Follow-up Invitation, such as utterances asking about the last content mentioned by the child (e.g. Tell me more about that), or about the content of events occurring after the last point in time mentioned by the child (e.g., Then what happened? ); Refocusing Invitation, such as utterances that refocused on previous content and request elaboration (e.g., Think back to the last time [or any other disclosed content], and tell me everything about that); and Closing Invitation such as closing utterances aiming for the child to disclose more information about the event (e.g., Is there anything else you remember about that day? ). Cued invitations were utterances that refocused the child’s attention on previously mentioned details and used them as contextual cues in open-ended invitations to elicit narrative free-recall responses. Refocusing could be related to content cues (e.g., activities, objects, people, actions) mentioned by the child (e.g., You mentioned [content mentioned by the child], tell me about that; Tell me everything that happened from [an occurrence/action mentioned by the child] until [another occurrence/action mentioned by the child]).

*Directive* questions referred to utterances that focused on event-related information mentioned by the child earlier in the interview and requested additional information (or clarification) using a category, mostly wh- questions (who, what, when, where, how). Directive questions were “cued recall” prompts (e.g., Where/when did it happen? What colour was the puzzle? ).

*Option-posing* prompts were closed-ended questions that focused the child’s attention more narrowly on aspects of the event. They tapped recognition memory processes and could be formulated as yes/no or forced-choice questions (e.g., Were the toys on the table when this happened? Were the toys inside the bag or on the table? ).

### Narrative Coherence

In the current study, the narrative coherence of children’s recall of the personally experienced live event was assessed based on key story grammar elements and temporal features included in the children’s reports. This coding scheme was developed by Brown and colleagues (2018) who adapted schemes introduced by Stein and Nezworski (1978), Peterson and McCabe (1991), and Kulkofsky et al. (2008). We only coded unique utterances, ignoring repeated information and information that was suggested or introduced by the interviewer. In each response, we searched for information pertaining to markers of narrative coherence, but because it was easy to confound the number of details reported with the type of story grammar elements relayed, each category was only coded once (present or absent) within an utterance.

In the story grammar framework (Stein & Nezworski, 1978), successful narratives include information about the context, content, and characters associated with an event. Thus, following Brown and colleagues (2018), we employed the categories: Setting (contextual details about the participants, social aspects, location and timing of the event), Initiating Event (the specific incident or action that set the event sequence into motion), Internal Response (details about the emotions, cognitions and goals of the characters), Attempt (the actions or efforts made by characters within the event to achieve a goal or respond to the initiating event). We also classified Simple Temporal Markers (simple descriptors of aspects of chronology, such as first, next, before, after) and Complex Temporal Markers (temporal indicators of conditional states, such as if, then, until, when). Additionally, we tailed the Descriptive Markers present in children’s responses (adjectives, adverbs, and modifiers, such as big, small, very, soon, only, just, almost, at first…). In their study, Brown et al. (2018) also included the categories Consequence (what happened after the event) and Reaction (feelings, thoughts, actions following the event). We chose to exclude the story grammar elements Consequence and Reaction from our coding scheme, as they were not relevant to the nature of the event remembered by the participants. This decision was based on ensuring the applicability and relevance of the coding framework to the specific context of our study. Table 1 presents the story grammar framework, outlining the key components of storytelling and providing examples for each.

**Table 1:** Story grammar framework: storytelling components and examples

Story telling component	Definition	Example of responses
Setting		
Protagonist	People at the event	“There was a woman”
Social	The profession/position of people	“She was a doctor”; “She was a researcher”
Physical	Where the event took place	“In a room at the hospital”; “In a small/big room”
Temporal	When the event took place	“About 10 o’clock”; “in the morning”; “in the afternoon”
Initiating event	How the event began	“We went in, she told me to play some games”
Internal response	Emotions, cognitions, goals	“She was nice”; “It was boring”; “We pretended to brush our teeth”
Attempt		
Activities of the event	Construction task; Make believe play; Joint Interactive Play; Demonstration Task; Description of a picture; Telling a story from a book; Cartoons; Conversation and reporting; Questions about: Emotions; Social Difficulties and Annoyance; Friends, Relationships and Marriage, Loneliness; Break; Creating a story.	“We looked at this picture”; “We made a puzzle”; “She asked me some questions”; “We played with some toys”
Description	Description about the content of the activities	“There was a cat and a pelican…”
Simple temporal markers	Simple descriptors of aspects of chronology	“first, next, then, before, after”
Complex temporal markers		
Conditional states	Temporal indicators of conditional states	“when, if/then, until”
Causal relations	Temporal indicators of causal relations	“because, so, in order to”
Optional states	Temporal indicators of optional states	“sometimes, usually, always, or, probably”
Descriptive Markers	Adjectives, adverbs, and modifiers	Adjectives: “pretty, big, small”; Adverbs: “very, extremely, quickly, soon, fully”; Modifiers: “only, just, almost, hardly, at first, at the end, simply”

The total story grammar elements were calculated as the sum of information reported in the Setting, Initiating Event, Internal Response, and Attempt categories. Only correct/accurate details were included in these calculations, ensuring the reliability of our analyses. Story grammar elements reported in response to each question type were counted separately and then combined into a total score. Dependent variables were calculated by dividing the cell count of interest (e.g., total number of story grammar elements elicited using invitations) by the appropriate grouping total (e.g., total number of invitations asked in the interview), for each child. This allowed us to explore whether different types of interviewer prompts were likely to elicit more story grammar elements, regardless of how many prompts of each type were posed during the interview.

Also in line with previous studies (Brown et al., 2018b; Reese et al., 2011), we computed four measures which capture commonly identified aspects of narrative coherence: *Chronology*, which comprised the sum of the information coded as Setting-Temporal, Simple Temporal Markers, and Complex Temporal Markers; *Content* which encompassed the sum of the information coded as Initiating Event and Attempt; *Context*, which included the sum of all Setting information, with the exception of Setting-Temporal scores (already included in the Chronology variable); and *Evaluation*, which comprised the sum of the information portraying Internal Responses.

### Reliability of Coding

The lead coder, who was blind to the children’s diagnosis and age, scored all 118 transcripts. An independent rater blind to the group membership of the children and to the aims and hypotheses of the research scored 24 randomly selected transcripts (20% of the total), which included transcripts relating to children of different ages and groups (Autistic and Non-autistic). Reliability was assessed throughout the duration of coding, and all disagreements were resolved by discussion. Cohen’s κ coefficients for agreement between raters indicated good to excellent reliability for all story grammar elements: Setting (κ = 0.90), Initiating Event (κ = 0.94), Internal Response (κ = 0.95), Attempt (κ = 0.95), Simple Temporal Markers (κ = 0.95), Complex temporal markers (κ = 0.90) and Descriptive Markers (κ = 0.95). The first coder’s ratings were used for all data analyses.

## Results

Discriminant function analyses revealed no significant effects for gender with respect to the story grammar elements included in children’s reports of the event, and thus, gender was not included in any of the analyses reported below.

### Analysis Plan

First, we examined the effects of delay and interviewer prompts on the markers of narrative coherence. Second, we analysed the effects of delay and interviewer prompt type on the narrative coherence scores, using the average number of story grammar elements recalled per question of each type. Research questions were analysed using a series of repeated measures Analyses of Variance (ANOVAs) with *Group* (autistic and non-autistic) as the between-subject factor and *Delay* (two weeks and two months) and *Prompt type* (invitations, directive, and option-posing) as the within-subjects factors. Data on children’s recall of the target event are presented separately for six categories: (a) *Total* number of story grammar elements; (b) *Chronology* (sum of information coded as Setting-Temporal, Simple Temporal markers, and Complex Temporal markers); (c) *Content* (sum of information about the Initiating Event and Attempt); (d) *Context* (sum of all Setting information, except Setting‐Temporal scores, which were included in the Chronology variable); (e) *Evaluation* (information describing Internal Response); and (f) *Descriptive Markers*. All parametric tests were conducted with child as the unit of analysis. When the assumption of sphericity was violated (Mauchly’s test), degrees of freedom were corrected using Greenhouse-Geisser estimates of sphericity. Effect sizes are indicated by partial eta squared (*ηp*2). Simple effects analyses (with Bonferroni corrections) were used to unpack significant interactions. All statistical comparisons were two tailed, using *p* <.05 as the level of significance.

### The Effects of Delay on Narrative Coherence

Table 2 shows means and standard deviations for the average number of story grammar elements recalled after the two-week and the two-month delays.

**Table 2:** Means and standard deviations for indices of narrative coherence in children’s recall, by group and delay

	Group and Delay													
	Total					Autistic					Neurotypical			
	2 W		2 M			2 W		2 M			2 W		2 M	
Indices of narrative coherence	*M*	*SD*	*M*	*SD*	Sig.	*M*	*SD*	*M*	*SD*	Sig.	*M*	*SD*	*M*	*SD*
Total number of indices	37.8	18.7	28.6	18.2	*	41.4	20.7	31.6	24.6		34.7	16.7	26.2	9.9
Chronology markers	12.9	7.9	9.2	7.2	*	13.0	8.1	10.2	9.5		12.9	7.7	8.3	4.2
Content markers	18.9	8.7	14.3	7.6	*	20.4	10.7	15.5	10.3		17.7	6.3	13.3	3.9
Context markers	7.3	5.3	5.7	5.9	*	7.9	5.1	6.3	6.3		6.9	5.4	5.2	3.6
Evaluation markers	2.2	2.1	1.6	1.9	*	2.5	2.1	1.8	2.5		1.9	2.0	1.5	1.4
Descriptive markers	6.8	6.3	5.4	4.9	*	9.0	8.1	6.6	6.4	*	5.0	3.5	4.4	2.8
*Notes.* 2 W: 2-week interview; 2 M: 2-month interview. * Significant delay difference *p* <.05														

First, a 2 (*Delay*: 2 weeks, 2 months) x 2 (*Group*: autistic and non-autistic children) repeated measures ANOVAs was carried out on the total number of story grammar elements spontaneously recalled by children. There was a significant main effect of *Delay*, *F* (1, 57) = 24.64, *p <*.001, *η*_*p*_*2* = 0.30. Overall, children recalled a significantly greater number of story grammar elements after two weeks than after two months. There was no significant main effect of *Group*, *F* (1, 57) = 1.89, *p* = .175, *η*_*p*_*2* = 0.03, and no significant *Group* x *Delay* interaction, *F* (1, 57) = 0.13, *p =*.723, *η*_*p*_*2* = 0.002.

We then carried out a series of 2 (*Delay*: 2 weeks, 2 months) x 2 (*Group*: autistic and non-autistic children) repeated measures ANOVAs on scores for each of the elements. There were significant main effects of *Delay* on the *Chronology*, *F* (1, 57) = 12.90, *p* <.001, *η*_*p*_*2* = 0.19, *Content*, *F* (1, 57) = 22.77, *p* <.001, *η*_*p*_*2* = 0.29, *Context*, *F* (1, 57) = 9.52, *p* = .003, *η*_*p*_*2* = 0.14, *Evaluation*, *F* (1, 57) = 4.93, *p* = .030, *η*_*p*_*2* = 0.08, and *Descriptive*,* F* (1, 57) = 5.10, *p* = .028, *η*_*p*_*2* = 0.08, category scores. There was also a significant main effect of *Group* on the *Descriptive* category scores, *F* (1, 57) = 6.04, *p* = .02, *η*_*p*_*2* = 0.10, but not overall nor for the other markers of narrative coherence, all *F*s < 1.89, *p*s > 0.175. There were no significant *Group* x *Delay* interactions, all *F*s < 2.12, *p*s > 0.152.

In sum, children’s reports included significantly more *story grammar* elements of all kinds after two weeks than after two months. Autistic children included significantly more *Descriptive* markers in their reports than non-autistic peers, but otherwise their reports were as coherent as those of their non-autistic peers.

## The Effects of Interviewer Prompt Type on Narrative Coherence

Table 3 shows means and standard deviations for the average number of story grammar elements recalled per question of each type (invitations, directive, and option-posing).

**Table 3:** Means and standard deviations for the average number of story grammar elements reported per question asked, by group and information type

	Group and Markers of Narrative Coherence																													
	Total					Chronology					Content					Context					Evaluation					Descriptive				
	Autistic		Neurotypical			Autistic		Neurotypical			Autistic		Neurotypical			Autistic		Neurotypical			Autistic		Neurotypical			Autistic		Neurotypical		
Prompt Type	*M*	*SD*	*M*	*SD*		*M*	*SD*	*M*	*SD*		*M*	*SD*	*M*	*SD*		*M*	*SD*	*M*	*SD*		*M*	*SD*	*M*	*SD*		*M*	*SD*	*M*	*SD*	
Invitations	1.12	0.50	1.20	0.50		0.44	0.29	0.55	0.32		0.71	0.29	0.78	0.26		0.29	0.19	0.30	0.23		0.08	0.09	0.09	0.08		0.29	0.21	0.23	0.15	
Directive	0.59	0.92	0.68	0.96		0.58	0.32	0.91	0.79		0.29	0.53	0.34	0.75		0.20	0.32	0.19	0.22		0.04	0.09	0.04	0.08		0.08	0.19	0.08	0.17	
Option-posing	0.29	0.25	0.27	0.27		0.12	0.19	0.05	0.08		0.17	0.12	0.17	0.16		0.07	0.09	0.07	0.11		0.04	0.07	0.02	0.04		0.07	0.10	0.05	0.09	

Initially, we carried out a 3 (*Prompt*: invitations, directive, option-posing) x 2 (*Group*: autistic, non-autistic children) repeated measures ANOVA on the total number of story grammar elements spontaneously recalled by the children. Degrees of freedom were corrected using Greenhouse–Geisser estimates of sphericity for the main effect of Prompt, ε = 0.71.

There was a significant main effect of *Prompt* for the total number of story grammar elements reported, *F* (1.4, 81.2) = 40.55, *p* <.001, *η*_*p*_*2* = 0.80. On average, invitations elicited significantly more story grammar elements than any other prompt type. Conversely, option-posing prompts elicited significantly fewer story grammar elements per prompt than any other prompt type. Directive prompts elicited significantly more story grammar elements per prompt than option-posing prompts did. There was no significant main effect of *Group*, *F* (1, 57) = 0.16, *p* = .687, *η*_*p*_*2* = 0.003, and no significant *Group* x *Prompt* interaction, *F* (1.4, 81.2) = 0.21, *p* = .738, *η*_*p*_*2* = 0.14.

We then carried out a series of 3 (*Prompt*: invitations, directive, option-posing) x 2 (*Group*: autistic, non-autistic children) repeated measures ANOVAs on scores for each of the elements. For analyses of the *Chronology scores*, degrees of freedom were corrected using Greenhouse–Geisser estimates of sphericity for the main effect of Prompt, ε = 0.68.

There was a significant main effect of *Prompt* for *Chronology*,* F* (1.4, 77.2) = 55.08, *p* <.001, *η*_*p*_*2* = 0.72. Directive prompts elicited significantly more chronological details per prompt than any other prompt type. Conversely, option-posing prompts elicited significantly fewer chronological details per prompt than any other prompt type. Invitations elicited significantly more chronological details per prompt than option-posing prompts did. There was no significant main effect of *Group*, *F* (1, 57) = 2.69, *p* = .107, *η*_*p*_*2* = 0.05, but there was a significant *Group* x *Prompt* interaction, *F* (1.4, 77.2) = 5.01, *p* = .019, *η*_*p*_*2* = 0.14. Following up using simple effects analyses revealed a significant *Group* effect for directive questions, *F* (1, 57) = 4.25, *p* = .044, *η*_*p*_*2* = 0.07. Pairwise comparisons (*p* <.05, with a Bonferroni correction) showed that autistic children reported significantly fewer chronological details per question in response to directive prompts than did age-matched non-autistic peers.

For analyses of the *Content scores*, degrees of freedom were corrected using Greenhouse–Geisser estimates of sphericity for the main effect of *Prompt*, ε = 0.63. There was a significant main effect of *Prompt*,* F* (1.3, 71.7) = 38.35, *p* <.001, *η*_*p*_*2* = 0.85. Invitations elicited significantly more content details per prompt than any other prompt type. Directive and option-posing prompts elicited a similar number of content details per prompt. There was no significant main effect of *Group*, *F* (1, 57) = 0.26, *p* = .614, *η*_*p*_*2* = 0.004, and no significant *Group* x *Prompt* interaction, *F* (1.3, 71.7) = 0.12, *p* = .790, *η*_*p*_*2* = 0.02.

In analyses of the *Context* scores, there was a significant main effect of *Prompt*,* F* (2, 114) = 24.32, *p* <.001, *η*_*p*_*2* = 0.53. Invitations elicited significantly more contextual details per prompt than any other prompt type. Conversely, option-posing prompts elicited significantly fewer context elements per prompt than any other prompt type. Directive prompts elicited significantly more contextual details per prompt than option-posing prompts did. There was no significant main effect of *Group*, *F* (1, 57) = 0.003, *p* = .954, *η*_*p*_*2* = 0.00, and no significant *Group* x *Prompt* interaction, *F* (2, 114) = 0.05, *p* = .0951, *η*_*p*_*2* = 0.002.

In analyses of the *Evaluation* scores, degrees of freedom were corrected using Greenhouse–Geisser estimates of sphericity for the main effect of *Prompt*, ε = 0.87, for which there was a significant main effect, *F* (1.7, 98.6) = 12.99, *p* <.001, *η*_*p*_*2* = 0.40. Invitations elicited significantly more evaluation details per prompt than any other prompt type. There were no other differences. There was no significant main effect of *Group*, *F* (1, 57) = 0.17, *p* = .683, *η*_*p*_*2* = 0.003, and no significant *Group* x *Prompt* interaction, *F* (1.7, 98.6) = 0.32, *p* = .693, *η*_*p*_*2* = 0.02.

Finally, in analyses of the *Descriptive* scores, degrees of freedom were corrected using Greenhouse–Geisser estimates of sphericity for the main effect of *Prompt*, ε = 0.81, for which there was a significant main effect, *F* (1.6, 90.7) = 30.66, *p* <.001, *η*_*p*_*2* = 0.50. Invitations elicited significantly more descriptive markers per prompt than any other prompt type. There were no other differences. There was no significant main effect of *Group*, *F* (1, 57) = 1.04, *p* = .311, *η*_*p*_*2* = 0.02, and no significant *Group* x *Prompt* interaction, *F* (1.6, 90.7) = 0.52, *p* = .556, *η*_*p*_*2* = 0.01.

## Discussion

In recent years, the understanding of recall abilities in autistic people has significantly advanced, particularly regarding their role as witnesses in criminal proceedings. Research has predominantly focused on the reliability of autistic witnesses’ testimony, exploring various techniques and procedures to enhance recall without compromising accuracy. Despite these developments, knowledge gaps remained, particularly regarding how coherently autistic children recall information about personally experienced events over time and respond to different types of interviewer prompts. This study uniquely bridged these gaps by exploring the coherence of narratives provided by autistic children in response to varying interviewer prompts, after two different delays, contributing novel insights into their recall capabilities.

This study demonstrated that intellectually and verbally capable autistic children, like their non-autistic peers, can recall experiences coherently after varying delays. As expected, both groups showed a decline in certain aspects of narrative coherence over time, which aligns with prior research on memory retention (e.g., Almeida et al., 2019a; La Rooy et al., 2005). Importantly, while the non-autistic group experienced a reduction in descriptive richness, autistic children displayed a specific reduction in chronological coherence, particularly in response to directive prompts. This observation suggests unique cognitive challenges in temporal processing among autistic children (e.g., Boucher et al., 2007; Jurek et al., 2019). Conversely, autistic children outperformed non-autistic peers in providing richer descriptive markers, offering rich details that enhanced the vividness and clarity of their narratives. This finding highlighted their ability to add clarity and detail, which is a vital aspect of coherent storytelling and an important consideration in understanding narrative abilities. As noted by Stein and Glenn (1979), children can make their accounts clear, organized, and understandable by including key elements such as adjectives, adverbs, and modifiers in their reports. However, caution is advised in generalizing these findings across the broader autism spectrum, particularly for individuals with additional cognitive or linguistic challenges. We cannot be certain that the same remarkable capabilities would be manifest by less intellectually and/or verbally competent autistic children, who might have poorer memories of the event.

Consistent with our hypotheses, our study found that autistic children were as adept as non-autistic children in providing detailed narratives about events they experienced, including in their reports information about the context, content, and characters associated with the event. This finding supported earlier reports that well-matched groups of autistic and non-autistic children have similar narrative abilities (e.g., Diehl et al., 2006; Henry et al., 2020; Losh & Gordon, 2014; Young et al., 2005) and underscored the ability of autistic children to be effective communicators in structured settings. Significantly, autistic children demonstrated a particular strength in incorporating *descriptive* markers into their narratives. This finding highlighted their ability to add clarity and detail, which is a vital aspect of coherent storytelling and an important consideration in understanding narrative abilities. As noted by Stein and Glenn (1979), children can make their accounts clear, organised, and understandable by including key elements such as adjectives, adverbs, and modifiers in their reports. However, caution is advised in generalizing these findings across the broader autism spectrum, particularly for individuals with additional cognitive or linguistic challenges. We cannot be certain that the same remarkable capabilities would be manifest by less intellectually and/or verbally competent autistic children, who might have poorer memories of the event.

Open-ended child-led recall prompts are known to elicit accurate accounts from vulnerable interviewees, including cognitively and verbally able autistic children (Almeida et al., 2019b; Brown et al., 2012, 2015, 2017; Brown & Lamb, 2015). Our findings thus added to previous research demonstrating the effectiveness of using such prompts to elicit semantically diverse and coherently structured narratives from them. Specifically, children in both groups were more likely to report information about the participants in the event, including about the emotions, cognitions, and goals of the characters; details about the location of the event, how the event began and the activities constituting the event, when questioned using open-ended prompts.

Compared to other types of prompts, directive questions elicited more information related to the chronology of the event from children in both groups, including contextual details about the timing of the event and indications of the order/sequence of actions and activities. These questions refocus children on previously mentioned details regarding the event, and vary widely in their specificity, eliciting information that children might not produce in response to invitations and cued invitations (Steward et al., 1996). Moreover, directive *wh*- prompts can effectively elicit information from younger children (Kulkofsky & Klemfuss, 2008b) and from autistic children (McCrory et al., 2007b), because they make specific requests that demand less retrieval effort. Professionals should make careful use of more specific directive questions, however, particularly in interviews after a lengthy delay, because *wh*- questions often elicit single-word or -phrase responses that are more likely to be inaccurate than information elicited using broader prompts (Brown et al., 2013).

In the present study, directive questions, while effective for eliciting chronological information, were less so for autistic children. The observation that autistic children provided fewer chronological details in response to directive prompts could indicate difficulties with temporal cognition as suggested in prior research (Boucher et al., 2007; Bowler et al., 2000; Jurek et al., 2019). The well documented difficulties these children have thinking diachronically may have resulted in poorer performance in response to open-ended directive prompts, even though the information provided in response to such questions by autistic people is usually as accurate as that provided by non-autistic peers (Almeida et al., 2019a, b; Boucher et al., 2007; Maras et al., 2013; Maras & Bowler, 2010, 2012). However, it is also possible that these questions are simply less effective when addressed to autistic children. These findings underscore the importance of tailored interviewing strategies in forensic settings, particularly for autistic children. Given their relative strength in providing descriptive detail, open-ended prompts should be prioritized to elicit rich, clear narratives, while directive prompts may need further adaptation to minimize confusion regarding temporal details.

Overall, our results demonstrated that autistic children could produce informative narratives, even after a delay of two months. While our study was adequately powered to detect the main effects, the exploration of more intricate interactions within the autistic group, such as how specific autism characteristics or comorbid conditions might influence narrative coherence, was limited by our sample size. Future research with larger and more diverse samples is necessary to investigate these complex interactions, which could provide critical insights into individual differences in narrative abilities among autistic children. Practical constraints prevented us from assessing verbal or full-scale IQ, but only autistic children and intellectual and linguistic abilities within the normal range (verbal quotients of 85 or above and full‐scale IQ of 90 or above) participated and non-autistic children did not have any symptomology or known intellectual, developmental, or neurological disorders. By focusing on cognitively and verbally able autistic children, our study provided valuable insights into the narrative capabilities intrinsic to autism, unconfounded by intellectual or linguistic impairments. This clarity is essential for developing targeted interventions and support strategies in legal contexts.

The constraints involved in conducting a staged event for an experimental study meant that the children were questioned about a neutral standardized event. The event used in the current study was rich, long, and interactive. However, as noted by Losh and Capps (2003) and highlighted by Henry and colleagues (2020), experimental findings may not reflect children’s narrative competence “within the less structured and more socially demanding contexts of daily life” (p. 248). Also, real criminal events are likely to be physically and/or emotionally stressful, so we cannot assume that the same memory capabilities would be observed in relation to such events.

In the present study, children were probed for information using a range of differently formulated recall and recognition-based prompts and questions, which reflect how forensic interviews might proceed. Like previous findings (Almeida et al., 2019a, b), our results have important implications for the legal realm because they provide further evidence that freely recalled information constitutes the richest form of testimony (Almeida et al., 2019c; Lamb et al., 2018), including from children on the autism spectrum. Nevertheless, empirical evidence suggests that autistic children typically demonstrate higher levels of anxiety than non-autistic children, particularly in unexpected situations, and may react negatively (emotionally, cognitively, and/or behaviourally) in uncertain contexts (e.g., Boulter et al., 2014). Encounters with the legal system are frequently distressing and are associated with poorer memory performance than in other contexts (e.g., Nathanson & Saywitz, 2003; Quas & Lench, 2007), therefore generalizing the current findings to stressful real-world events must be done with caution.

In sum, the current study revealed that cognitively and verbally able autistic children can convey their individual experiences in a structured, organized, and coherent manner, when appropriately questioned, even after lengthy delays. The present findings also provided further evidence for the task support hypothesis (Bowler et al., 1997) demonstrating that the best-practice principles embodied in the Revised NICHD Protocol effectively elicit coherent narratives from cognitively and verbally able children on the autism spectrum. Because literal and concrete thinking is common in autistic people, communicating expectations clearly and motivating children to provide as much information as they can throughout the interviews might have influenced their performance by underlining the unique demands of the interview context (Sternberg et al., 2002).

Governments and societies have come a long way toward recognising the vulnerabilities and needs of individuals with developmental disorders and in many countries have made it a priority to address those needs (e.g., Durcan et al., 2014; Gerry et al., 2022; Ministry of Justice, 2023; National Disability Authority, 2018). Many advances have been made to raise public awareness of autism but despite these advances professionals still have difficulty implementing best-practice recommendations and making the adaptations needed to elicit best evidence from autistic people (Crane et al., 2016; Slavny-Cross et al., 2022). Many professionals have recognized their vulnerabilities and expressed the need for specialized training, information, and more organisational support (Crane et al., 2016), which is crucial to ensure that professionals respond effectively and fairly to autistic people within the legal system. In fact, recent studies (Crane et al., 2020; Maras et al., 2019) found that explicitly inform jurors of witnesses’ autism diagnoses, alongside provision of further specific information about each autistic individual, resulted in a positive bias regarding witnesses’ perceived credibility. These findings highlight the importance of carefully developing educational resources for legal professionals, tailored to individual children, outlining how their autism manifests and how/if it might affect their testimony (Crane et al., 2020), to ensure that the credibility of autistic children is not unfairly undermined. Legal professionals need appropriate training to be able to fully comprehend the myriad manifestations of autism and to ensure that investigative practices are evidence-based.
